# Community engagement and data quality: best practices and lessons learned from a citizen science project on birdsong

**DOI:** 10.1007/s10336-022-02018-8

**Published:** 2022-10-13

**Authors:** Denise Jäckel, Kim G. Mortega, Sarah Darwin, Ulrich Brockmeyer, Ulrike Sturm, Mario Lasseck, Nicola Moczek, Gerlind U. C. Lehmann, Silke L. Voigt-Heucke

**Affiliations:** 1 Museum für Naturkunde Berlin, Leibniz Institute for Evolution and Biodiversity Science, Berlin, Germany; 2 Life Sciences Faculty, Humboldt-Universität zu Berlin, Berlin, Germany; 3 PLAN Institute for Architectural and Environmental Psychology, Berlin, Germany; 4 Evolutionary Ecology, Department of Biology, Humboldt-Universität zu Berlin, Berlin, Germany; 5 Animal Behaviour, Institute of Biology, Freie Universität Berlin, Berlin, Germany

**Keywords:** Community science, Public engagement, COVID-19, Pandemic, Birdsong, Dialect

## Abstract

Citizen Science (CS) is a research approach that has become popular in recent years and offers innovative potential for dialect research in ornithology. As the scepticism about CS data is still widespread, we analysed the development of a 3-year CS project based on the song of the Common Nightingale (*Luscinia megarhynchos*) to share best practices and lessons learned. We focused on the data scope, individual engagement, spatial distribution and species misidentifications from recordings generated before (2018, 2019) and during the COVID-19 outbreak (2020) with a smartphone using the ‘Naturblick’ app. The number of nightingale song recordings and individual engagement increased steadily and peaked in the season during the pandemic. 13,991 nightingale song recordings were generated by anonymous (64%) and non-anonymous participants (36%). As the project developed, the spatial distribution of recordings expanded (from Berlin based to nationwide). The rates of species misidentifications were low, decreased in the course of the project (10–1%) and were mainly affected by vocal similarities with other bird species. This study further showed that community engagement and data quality were not directly affected by dissemination activities, but that the former was influenced by external factors and the latter benefited from the app. We conclude that CS projects using smartphone apps with an integrated pattern recognition algorithm are well suited to support bioacoustic research in ornithology. Based on our findings, we recommend setting up CS projects over the long term to build an engaged community which generates high data quality for robust scientific conclusions.

**Supplementary Information:**

The online version contains supplementary material available at 10.1007/s10336-022-02018-8.

## Abstract (de)

**Gesellschaftliches Engagement und Datenqualität: Bewährte Praktiken und Erfahrungen aus einem bürgerwissenschaftlichen Projekt zum Vogelgesang**

Citizen Science (CS) ist eine Forschungsmethode, die in den letzten Jahren an Bedeutung gewonnen hat und innovatives Potenzial für die Dialektforschung in der Ornithologie bietet. Da die Vorbehalte gegenüber CS-Daten immer noch weit verbreitet sind, haben wir die Entwicklung eines dreijährigen CS-Projekts zum Gesang der Nachtigall (*Luscinia megarhynchos*) analysiert, um bewährte Praktiken und gewonnene Erfahrungen darzustellen. Wir fokussierten uns auf den Datenumfang, das individuelle Engagement von Teilnehmenden, die räumliche Verteilung und die Fehlbestimmungen von Arten aus Aufnahmen, die vor (2018, 2019) und während des COVID-19-Ausbruchs (2020) mit einem Smartphone unter Verwendung der "Naturblick" App erstellt wurden. Die Anzahl der Aufnahmen von Nachtigallgesängen und das individuelle Engagement stiegen stetig an und erreichten ihren Höhepunkt in der Saison während der Pandemie. 13.991 Aufnahmen von Nachtigallgesängen wurden von anonymen (64%) und nicht-anonymen Teilnehmenden (36%) erstellt. Im Laufe des Projekts weitete sich die räumliche Verteilung der Aufnahmen aus (von Berlin auf bundesweit). Die Rate der Fehlbestimmungen war gering, ging im Laufe des Projekts zurück (von 10% auf 1%) und wurde hauptsächlich von gesanglichen Ähnlichkeiten mit anderen Vogelarten beeinflusst. Unsere Studie zeigte außerdem, dass das gesellschaftliche Engagement und die Datenqualität nicht direkt von den durchgeführten Disseminationsaktivitäten beeinflusst wurden, sondern dass erstere von externen Faktoren abhingen und letztere von der App profitierte. Wir schließen daraus, dass CS-Projekte, die Smartphone-Apps mit einem integrierten Mustererkennungsalgorithmus verwenden, gut geeignet sind, um die bioakustische Forschung in der Ornithologie zu unterstützen. Auf der Grundlage unserer Ergebnisse empfehlen wir, CS-Projekte langfristig zu etablieren, um eine aktive Teilnehmergemeinschaft (Community) aufzubauen, die qualitativ hochwertige Daten für fundierte wissenschaftliche Schlussfolgerungen generiert.

## Introduction

Citizen science (CS) is a research approach in which volunteers (non-professional scientists) and academic researchers (professional scientists) work together in one or several research processes to gain scientific knowledge (Bonney et al. 2014). In recent years, CS has been increasingly used in ecological and environmental research (e.g., Planillo et al. 2021). With the development of technical tools for citizen scientists such as interactive platforms or smartphone apps, the data collection increased recently (Falk et al. 2019). This technological improvement expanded local (e.g., Urban Birds Conservation Program of Vitoria-Gasteiz) and national projects (e.g., Dialects of Czech Yellowhammers: Diblíková et al. 2019) to global ones with real time data transmission (e.g., Xeno-Canto, Cornell Lab of Ornithology). Citizen scientists generated data on a large temporal and spatial scale that could otherwise not be obtained (Diblíková et al. 2019; Searfoss et al. 2020). The aim of CS studies can be quite diverse, e.g., they can serve conservation, raise awareness or monitor environmental efforts (e.g., Roy et al. 2012; Planillo et al. 2021). In the field of ornithology, CS has a long tradition and contains some long-running projects; for example, the National Audubon Society’s Christmas Bird Count, the North American Breeding Bird Survey and the Pan-European Common Bird Monitoring Scheme. The number of long-term projects has increased ever since, while professional and lay bird enthusiasts are continuing to upload data on online platforms and archives (e.g., eBird: Sullivan et al. 2009, ornitho.de: Frick and Jaehne 2013, Project FeederWatch). Indeed, Randler (2021) showed that people using ornitho.de have better birding skills compared to other birders. Citizen scientists with a longer engagement provide a large amount of valid data e.g., to document the worldwide presence or absence of birds (Lepczyk al. 2005).

Ecological estimates of diversity or abundance based on CS data can be affected by project structure and thus community engagement as well as data quality (Dickinson et al. 2010). Community engagement, in the sense of generated data, have been found to decrease with project duration (Bruckermann et al. 2021). In contrast, the COVID-19 outbreak starting in 2020 increased individual engagement and data scope in CS projects (Hochachka et al. 2021). The pandemic also changed human activities around the world in terms of data distribution, probably due to a greater desire to spend time in nature (Venter et al. 2021), and quality (Phillips et al. 2021). Data quality is multidimensional and may be expressed by more than a dozen factors (reviewed in Lewandowski and Specht 2015) such as anonymity or inexperience of citizen scientists and project duration (Dickinson et al. 2010). In bird studies, this can lead to misidentifications. Identification is a complex task that relies on several factors such as vocal and visual similarities between co-occurring species or species richness (Kelling et al. 2015).

We here aimed to gain a better understanding of the community engagement and data quality in a CS project that was based on the song of the Common Nightingale (*Luscinia megarhynchos*) before (2018, 2019) and during the COVID-19 pandemic (2020). The nightingale is an ideal study object to address scientific questions using a CS approach. Nightingales possess a very memorable melodic and complex song which can frequently be heard from mid-April to late June in parks and gardens (Glutz von Blotzheim 1988) in Berlin, Germany. The literal translation in German ('*Nacht*'igall) as well as in English ('*night*'ingale) is a "night singer" and suggests a purely nocturnal song. Yet, nightingales are known to sing during both the night and the day-time (Amrhein et al. 2004). In the course of three nightingale breeding seasons, we invited all citizens to record nightingales with their smartphones. Based on previous CS projects which showed that difficult tasks reduce data quality (Kosmala et al. 2016), we decided against strict protocols or specific instructions (duration, number, place or time of the recordings). Instead, we chose a low-threshold approach to target citizen scientists with little or no prior ornithological knowledge. The intent was to reach a wide audience, resulting in a diverse, high level of community engagement. The project, which started in Berlin in 2018, has been expanded to cover all of Germany from 2019 to 2020. We allowed the participants to engage anonymously or non-anonymously. As it has been shown that many dissemination activities increase data quality (Bryant and Oliver 2009), we aimed to provide information about nightingale song on the project website, in press coverage, and at scientific or cultural face-to-face events, mostly in Berlin and mainly before the COVID-19 pandemic.

The nightingale citizen science project (*Forschungsfall Nachtigall*) was based on a 2016 pilot project and previous dialect findings in the nightingale song (master thesis: Schehka 2004; doctoral thesis: Weiss 2012). Dialects are song variations between different populations and time periods (Catchpole and Slater 2008). For dialect studies, many recordings with a wide spatial distribution are needed. A growing body of literature demonstrates that this can easily be obtained through the power of worldwide participating citizen scientists (e.g., Diblíková et al. 2019; Searfoss et al. 2020). Our opportunistic approach indeed led to a large collection of geo-referenced nightingale song recordings. We previously showed that the majority of our CS data were valid enough (Jäckel et al. 2021) and of high value for dialect studies (Jäckel et al. 2022). The development of the project over three nightingale breeding seasons has not yet been studied.

Here, we thus focused on the community engagement (data scope, spatial distribution) and data quality (species misidentifications) before and during the COVID-19 pandemic. We investigated (1) the data scope in terms of the number of participants, cumulative duration and number of recordings from participants who took part either anonymously or non-anonymously, (2) the spatial and temporal distribution of recordings, and (3) species misidentifications in total and from anonymous users and non-anonymous ones and underlying patterns. In 2020 during the COVID-19 pandemic, we predicted to find a decrease in community engagement due to our reduced dissemination activities, yet an increase in individual engagement. The project has been promoted with a wider geographic outreach after the first year, whereby we expected that recordings would be more widely distributed over the last 2 years. We predicted that particularly common and other melodious bird species were mistaken for nightingales—especially during the night—as lay people often assume that only nightingales have a nocturnal song.

## Methods

### The nightingale citizen science project and its cooperation with the ‘Naturblick’ app

The nightingale citizen science project was launched in 2018 as a collaboration with the 'Naturblick' app at the Museum für Naturkunde (MfN) in Berlin, Germany. The app has been available since 2016 and has already been widely used in 2017 with almost 50,000 downloads (Sturm and Tscholl 2019). As a special feature, the app includes a pattern recognition algorithm (PRA) which automatically identifies bird species based on cross-correlation via template matching of spectrogram segments (Lasseck 2016; Stehle et al. 2020). During the last years, the PRA improved due to the use of neural networks as well as deep learning (Lasseck 2018).

**Fig. 1:**
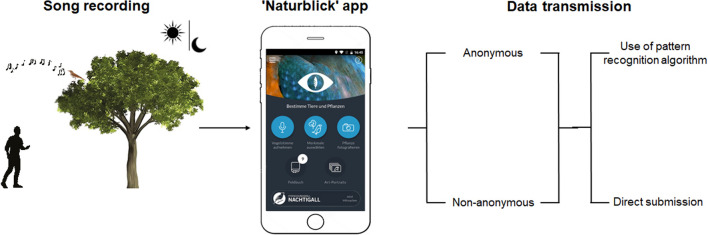
Left: Process of song recording transmission. Middle: Display of the ‘Naturblick’ app. Right: For data transmission, users could participate anonymously or non-anonymously and either make use of the pattern recognition algorithm or directly submit the recording

Using diverse dissemination activities such as events both inside and outside the MfN, midnight excursions and press coverage, the public was invited to download the 'Naturblick' app on their smartphones and record nightingale songs (for details see Jäckel et al. 2021). Public events were free of charge, included two to 180 participants and by large took place in Berlin. Press coverage occurred mainly before and during the breeding season in the form of radio interviews, newspaper articles and social media posts. The app featured a bespoke button showing a nightingale, to highlight the citizen science project. By clicking on this button, participants could transmit their recordings directly or make use of the PRA to aid in species identification (Fig. 1). As part of this process, the three bird species whose vocalisations most closely match the recording were presented (Stehle et al. 2020). Participants were also allowed to choose whether they wanted to submit the recordings anonymously or non-anonymously with an individual username. Because of technical limitations, it was not possible to submit recordings more than once or with a duration of more than two minutes per recording. Temporal (day, time) and spatial (GPS coordinates) information were automatically captured in the metadata, if permitted by the participant.

### Development of the data scope over the study period

To get a better understanding of the community development over the course of the project, we descriptively analysed the data scope before (2018, 2019) and during the COVID-19 pandemic (2020). We used the number of participants, cumulative duration and number of all recordings by participants who took part anonymously or non-anonymously in 1, 2, or 3 years as parameters. As dissemination activities are supposed to have an impact (Bryan and Oliver 2009), we examined whether their reduction in 2020 had any effect. Less dissemination activities were provided during the 2020 nightingale breeding season. That year was dominated by the COVID-19 pandemic lockdown and face-to-face events were not permitted in Berlin. Instead, online field trips and events were undertaken, a method which has been found to be equally reliable (Rögele et al. 2022).

### Development of spatial distribution of CS recordings in Germany

To enable comparisons between populations, it is important to obtain recordings from different places. Citizen scientists, therefore, have to cover a wide spatial distribution. We aimed to reach people from Berlin, where the MfN is based, and all over Germany, since participation in this project was possible from any location with a smartphone and the 'Naturblick' app. Germany was separated into four regions (North, East, South, West) which included quadrants with an area of 100 × 100 kms each. We divided the number of quadrants that contained nightingale song recordings by the number of total quadrants in Germany to determine the percentages for each year and whether the spatial distribution increased with time.

### Underlying patterns of species misidentifications

Species identification is challenging for citizen scientists (Crall et al. 2011) and one of the major data quality issues. We aimed to elucidate and better understand the development of species misidentifications over the course of the project and underlying patterns. MP3 and m4a files were converted into the WAV format using the WaveLab 7 program to analyse them visually and acoustically with Avisoft SASLab Pro 5.2 (R. Specht, Berlin, Germany, sampling rate = 22.050 Hz, FFT = 1024 points, Hamming-Window, overlap 93.75%). Recordings were sorted into four types (nightingale songs, nightingale calls, other bird species, i.e., species misidentifications of the nightingale and no bird song). We examined the effect of dissemination activities in all three nightingale breeding seasons and of participation (i.e., anonymity, experience) on the recording types and descriptively analysed the respective percentages (number of recordings per classified type/all recordings).

To identify temporal factors for underlying patterns (calendar week and time of day), we descriptively compared the number of nightingale songs and other bird species recorded across all study years. The peak singing time of nightingales (23:00–1:00 h) as reported in the literature (see Jäckel et al. 2021) and other species of birds (5:00–7:00 h and 19:00–21:00 h) were accounted for to see if they have an influence on the rate of species misidentifications. To understand if and when certain species are most often misidentified as nightingales, we compared recordings from day (04:00–22:00 h) and night (22:00–04:00 h). Not all of the other bird species could be identified (too short, disturbing background noise).

As there were few species misidentifications in total (about 1–10% of submissions), we decided to work on the taxonomic group level. We analysed underlying patterns in terms of similarities (vocal, visual and species abundance) between the taxonomic group of the other bird species and the nightingale. Vocal similarities were determined by the following parameters: melodic (yes/no), complex (yes/no), nightingale song elements (whistle/trill/buzz) and usual time of singing (night/dawn/day). Nightingales are visually recognisable on their song post. Thus, we determined for the evaluation of visual similarities, whether the plumage was identical to the nightingale (brown), similar (black) or different. As frequently occurring species are easier to identify than rarely occurring ones (Falk et al. 2019), we determined the species abundance in Germany from the ‘Berliner Ornithologische Arbeitsgemeinschaft e.V.’ (Berlin Ornithological Society) breeding bird monitoring (http://www.orniberlin.de/) and divided it into six groups (group 1 =  ≤ 1,000, group 2 =  ≤ 10,000, group 3 =  ≤ 100,000, group 4 =  ≤ 500,000, group 5 =  ≤ 1,000,000, group 6 =  > 1,000,000).

### Statistical analyses

We performed all statistical analyses with R version 4.1.2 (R-Team 2021). A possible correlation between (i) the dissemination activities and the number of participants, (ii) cumulative duration and the recording type (iii) or number of recordings and the recording type was determined using Spearman's rank correlation test. We used a principal component analysis (PCA) and a general linear model (GLM) to compare similarities (vocal, visual and species abundance) between taxonomic groups and nightingales.

## Results

### Development of the data scope over the study period

**Fig. 2:**
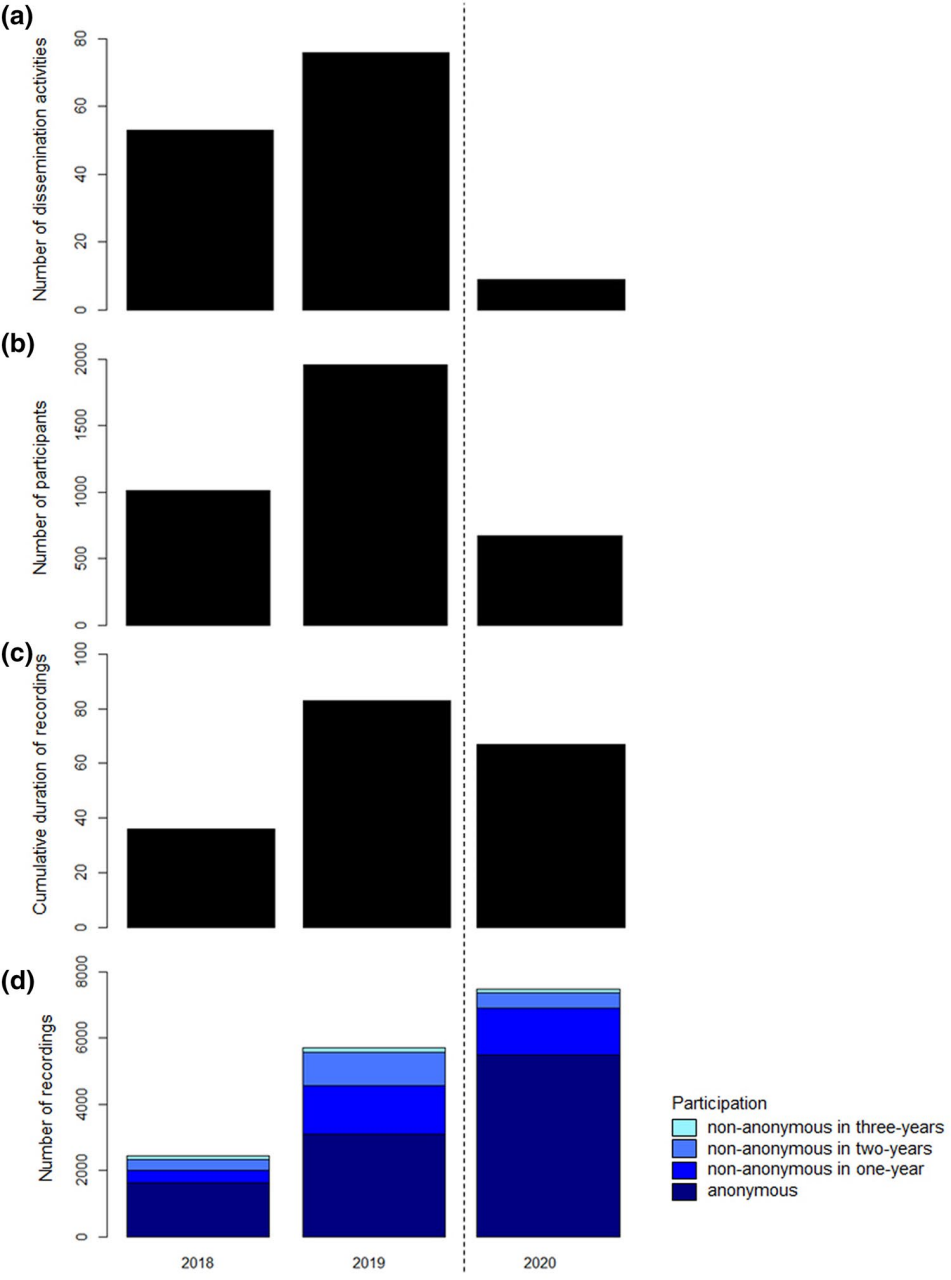
Overview of the project's data scope development with regard to the total number of dissemination activities (**a**), number of participants (**b**), cumulative duration of recordings (**c**), and number of recordings from citizen scientist who participated anonymously or non-anonymously in 1, 2, or 3 years (**d**). The dashed line separates the years before (2018, 2019) and during (2020) the COVID-19 pandemic

The project showed a positive community development over the project duration in terms of the data scope. The number of dissemination activities (Fig. 2a, Online Resource 1), cumulative duration (Fig. 2b) and number of participants (Fig. 2c) increased from 2018 to 2019 and decreased in 2020. Number of recordings increased continuously over the nightingale breeding seasons (Fig. 2d), this was due to a higher number of individual engagement especially in 2020. The majority of data were recorded anonymously, followed by citizen scientists who participated non-anonymously in 1, 2 and 3 years. Dissemination activities did not correlate with the number of participants, cumulative duration or number of recordings (Spearman's rank correlation test: *p* > 0.05).

### Development of spatial distribution of CS recordings in Germany

**Fig. 3:**
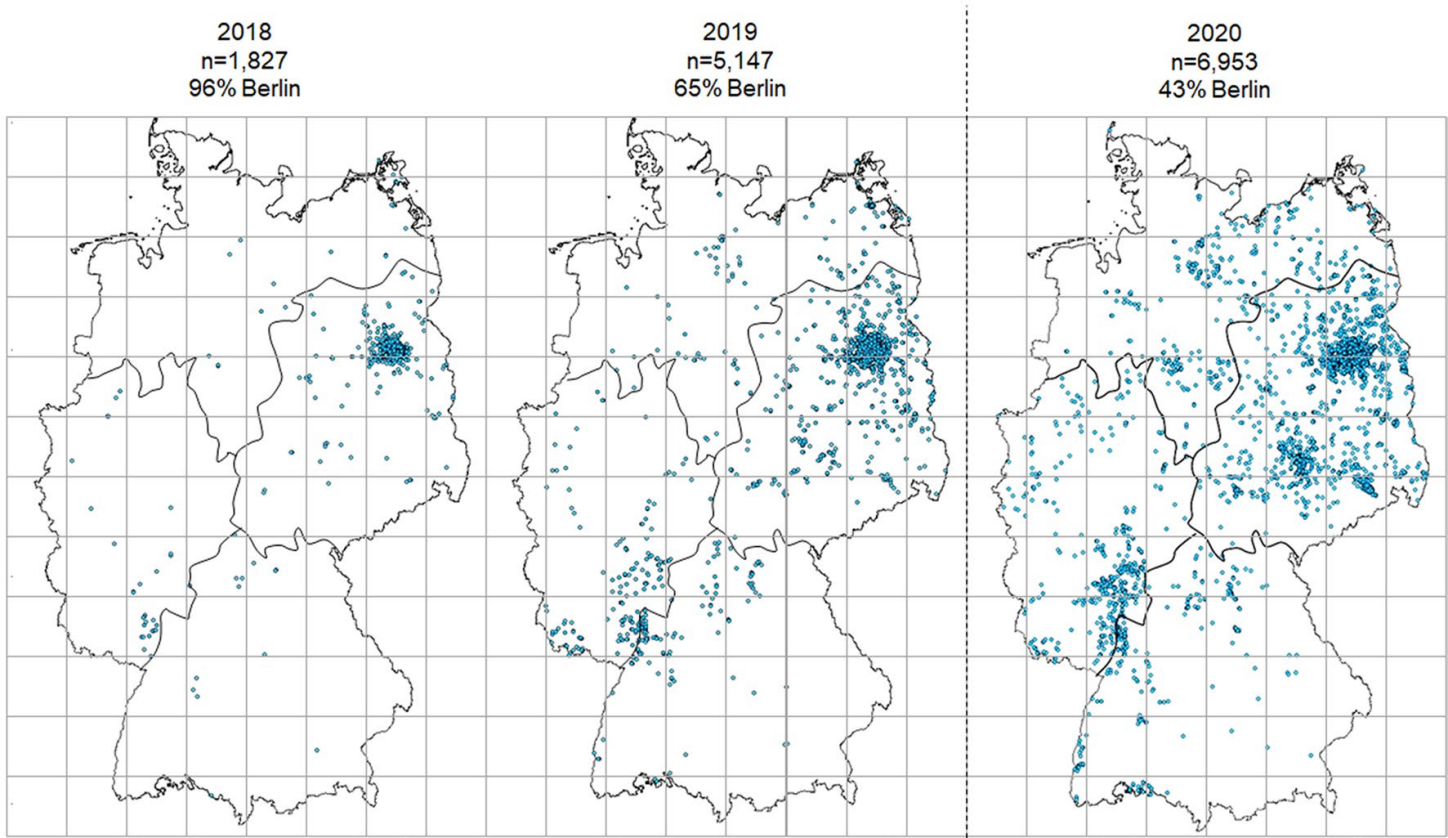
The spatial distribution of nightingale song recordings in the CS project from Germany. The dashed black line separates the years before (2018–2019) and during the COVID-19 pandemic (2020). Black continuous lines show the four regions (North, East, South, West). Grey solid lines represent quadrants (100 × 100 kms)

Most recordings were obtained from the East of Germany, followed by the West, North and South (Fig. 3). This matches the natural distribution of the nightingale (reported by the German national bird breeding count by the ‘Dachverband Deutscher Avifaunisten; Gedeon et al. 2014). During 2018, we had the lowest spatial distribution in terms of the percentage of quadrants which contained nightingale song recordings (42%). In 2020, during the COVID-19 pandemic, the spatial distribution for all regions was larger (90%) than in 2019 (74%). Over the years, the data were more geographically spread and no longer came mainly from Berlin as in the first year.

### Underlying patterns of species misidentifications

**Fig. 4:**
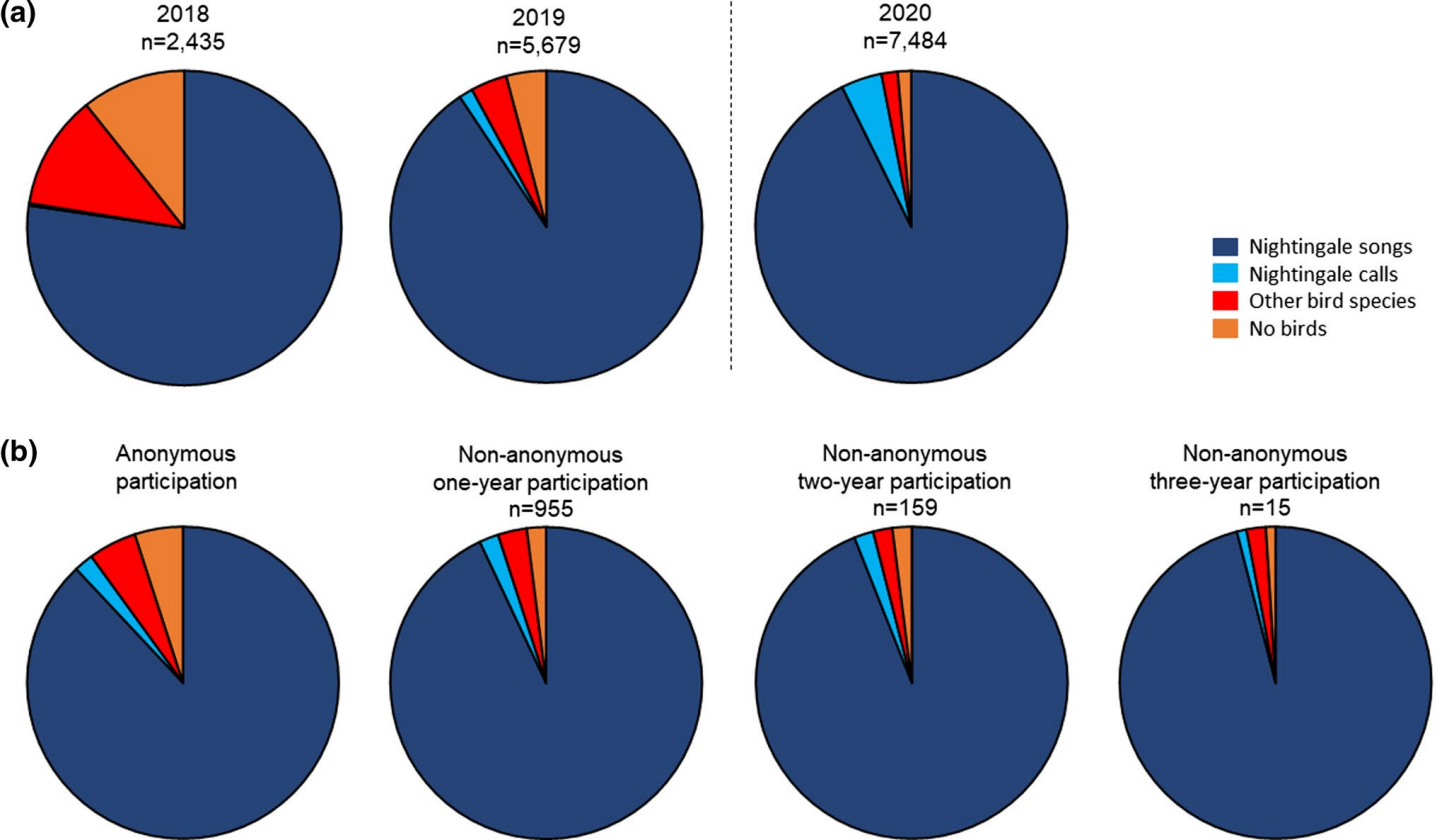
Comparison of CS recordings with regard to percentages of recording types (**a**) and from citizen scientists who participated anonymously or non-anonymously (**b**). The dashed line separates the years before (2018, 2019) and during (2020) the COVID-19 pandemic

The data quality of the recording types showed a positive development over the 3 years. The percentage of nightingale songs and calls (Fig. 4a) increased steadily over the study period. At the same time, the percentage of other bird species and no birds decreased continuously. In 2020, during the COVID-19 pandemic, the number of species misidentification was the lowest. Dissemination activities did not correlate with the recording types (Spearman's rank correlation test: *p* > 0.05). The percentages were similar between anonymous and non-anonymous participants (Fig. 4b, Online Resource 2). The highest percentage of recordings with nightingale songs and the fewest with other bird species were generated by non-anonymous citizen scientists who took part in all 3 years, followed by those who attended 2 years, 1 year and anonymously.

**Fig. 5:**
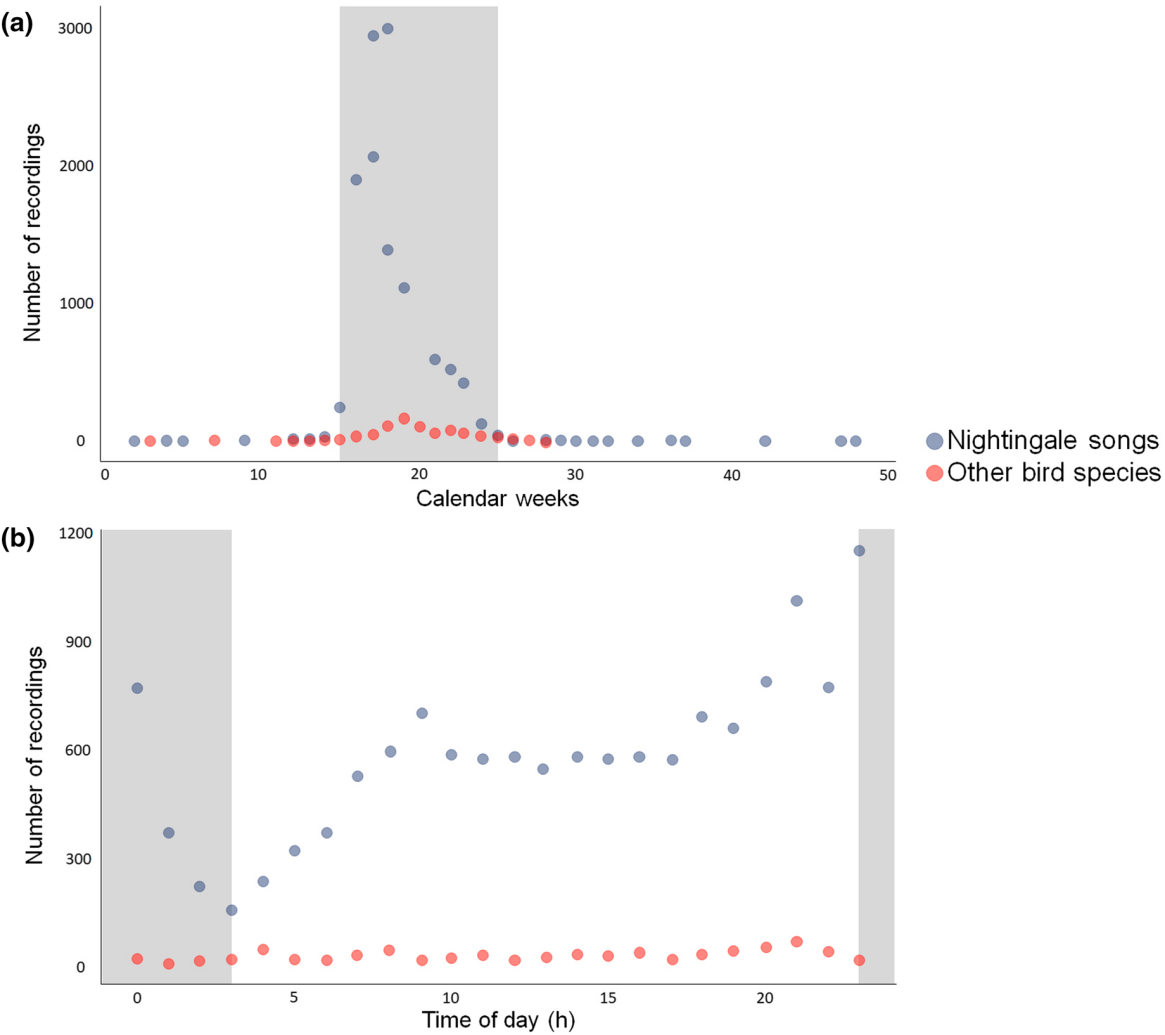
The temporal distributions of CS recordings for 13,991 nightingales and 601 other bird species (i.e., species misidentifications) during the whole study period (2018–2020). In grey, the periods of nightingale song as reported in the literature is highlighted. **a** The weekly number of recordings in the course of the breeding season. **b** The number of recordings in relation to the time of day (h)

A descriptive analysis of recordings from all years revealed that species misidentification was not affected by the song timing across calendar weeks or time of day. Most nightingale songs were recorded in the 16th and 17th calendar week (Fig. 5a). Recordings with other bird songs were made primarily between the 18th and 20th calendar week (see Jäckel et al. 2021). After the high nightingale breeding season when song is most common, slightly more (26th week) or the same amount (27th week) of other bird songs were recorded. Most recordings of nightingale song were made between 23:00 and 0:00 h and of other bird species at 21:00 and 4:00 h (Fig. 5b). There were more recordings with species misidentifications of other bird species recorded at hours outside the times when the nightingale song is regularly found (23:00 and 1:00 h).

During the day, European Blackbirds (*Turdus merula*, *n* = 295), Song Thrush (*Turdus philomelos*, *n* = 35) and House Sparrow (*Passer domesticus*, *n* = 31) were the species that were mainly misidentified as nightingales by participants (Table 1). European Blackbirds (*n* = 30), song thrush (*n* = 12) and European Robin (*Erithacus rubecula*, *n* = 5) were the species mainly misidentified at night. The Thrush Nightingale (*Luscinia luscinia*), the common nightingale's sister species (Vokurková, et al. 2013) was recorded fourteen times.

**Table 1:** The number of species misidentifications on the species level for 601 recordings of other bird species during the whole study period (2018–2020)

Species		Taxonomic group	Number of recordings	
English name	Latin name		Day	Night
European blackbird	*Turdus merula*	Muscicapoidea	295	30
Song thrush	*Turdus philomelos*	Muscicapoidea	23	12
European robin	*Erithacus rubecula*	Muscicapoidea	22	5
Common redstart	*Phoenicurus phoenicurus*	Muscicapoidea	10	1
Thrush nightingale	*Luscinia luscinia*	Muscicapoidea	8	6
European starling	*Sturnus vulgaris*	Muscicapoidea	4	0
Northern mockingbird	*Mimus polyglottos*	Muscicapoidea	2	0
Redwing	*Turdus iliacus*	Muscicapoidea	1	0
Bicknell’s thrush	*Catharus bicknelli*	Muscicapoidea	1	0
Black redstart	*Phoenicurus ochruros*	Muscicapoidea	1	1
House sparrow	*Passer domesticus*	Passeroidea	31	0
Common chaffinch	*Fringilla coelebs*	Passeroidea	13	2
European greenfinch	*Chloris chloris*	Passeroidea	4	0
European goldfinch	*Carduelis carduelis*	Passeroidea	2	0
House finch	*Haemorhous mexicanus*	Passeroidea	1	1
European serin	*Serinus serinus*	Passeroidea	1	0
Grey wagtail	*Motacilla cinerea*	Passeroidea	1	0
Icterine warbler	*Hippolais icterina*	Passeroidea	1	0
Pine grosbeak	*Pinicola enucleator*	Passeroidea	1	0
Eurasian blackcap	*Sylvia atricapilla*	Sylvioidea	26	1
Great tit	*Parus major*	Sylvioidea	10	0
Great reed warbler	*Acrocephalus arundinaceus*	Sylvioidea	4	2
Willow warbler	*Phylloscopus trochilus*	Sylvioidea	3	0
Eurasian blue tit	*Cyanistes caeruleus*	Sylvioidea	2	1
Western bonelli’s warbler	*Phylloscopus bonelli*	Sylvioidea	1	0
Arctic warbler	*Phylloscopus borealis*	Sylvioidea	1	0
Garden warbler	*Sylvia borin*	Sylvioidea	1	1
Eurasian wren	*Troglodytes troglodytes*	Certhioidea	11	0
Eurasian treecreeper	*Certhia familiaris*	Certhioidea	1	0
Carrion crow	*Corvus corone*	Corvoidea	4	0
Eurasian magpie	*Pica pica*	Corvoidea	1	0
Western jackdaw	*Corvus monedula*	Corvoidea	1	0
Eurasian scops owl	*Otus scops*	Strigidae	1	0
Common wood pigeon	*Columba palumbus*	Columboidea	2	0
European green woodpecker	*Picus viridis*	Picoidea Vigor	2	0

**Fig. 6:**
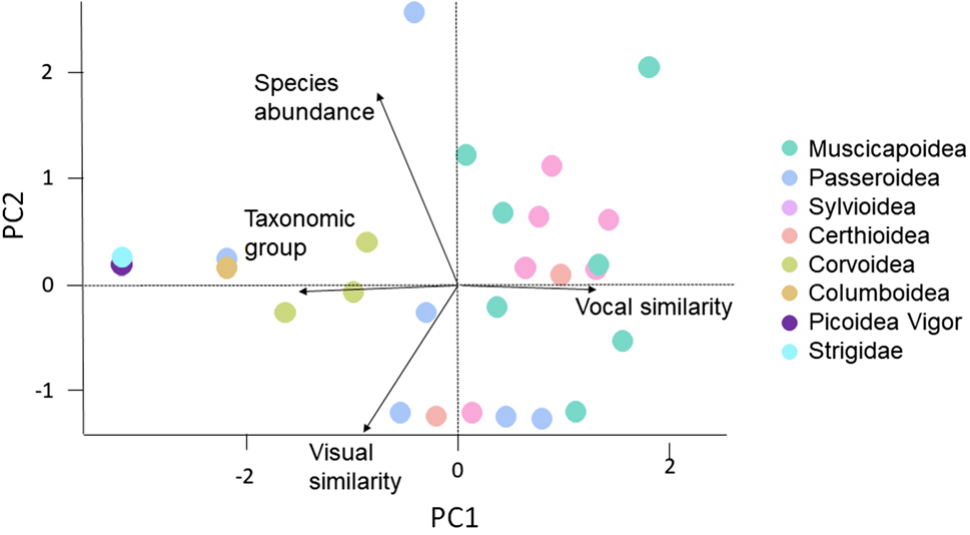
A principal component analysis (PCA) for 601 other bird species recordings during the whole study period (2018–2020). We compared similarities (vocal, visual and species abundance) between taxonomic groups and nightingales. The arrow length indicates the degree of similarities, the arrow direction indicates the association of the factors with the principal components PC1 and PC2

A PCA of three similarities traits (vocal, visual and species abundance) showed patterns of species misidentifications. Two principal components explained 71% of the species misidentifications with other species for vocal and visual similarities (Fig. 6, PC1 = 44%) and species abundance (PC2 = 28%) between the taxonomic groups and the nightingale. Vocal similarities mainly in the Muscicapoidea and Sylvioidea and partly in Certhiodea may have had an influence (for an overview of the species see Table 1). Visual similarities of Passeroidea and Certhiodea may have had an effect. Species abundance may be a factor for Passeroidea. A general linear model of the first principal component showed that species misidentifications on the level of the taxonomic groups were influenced significantly by the vocal similarity (GLM: *df* = 24, *p*-value < 0.005; Tab. 2).

**Table 2:** Results of a general linear model testing whether the first principal component (PC1) showed relationships between the traits and the taxonomic group

	Estimate	SEM	*t*	*p*
Taxonomic group	4.3249	1.6256	2.660	0.137*
Vocal similarity	– 0.613	0.1769	– 3.467	0.0020**
Visual similarity	0.5638	0.3200	1.762	0.0908
Species abundance	– 0.2869	0.1998	1.436	0.1639

## Discussion

In this paper, we investigated the development of community engagement and data quality of a 3-year CS project based on the song of the nightingale before and during the COVID-19 pandemic. We found an increase in the dynamics of community engagement over the course of the project in both the data scope and spatial distribution. The number of species misidentifications decreased during the study period and species misidentifications were mostly affected by vocal similarities of other species. In the following, we will discuss our findings in more detail with regard to two aspects: best practices and lessons learned that we believe could be useful to grow and improve (future) avian-based CS projects.

### Best practises: success factors in citizen science

The nightingale CS project steadily grew in numbers of recordings collected, despite all the challenges that CS brings (Bonney et al. 2014; Wittman et al. 2019) and the COVID-19 pandemic brought in 2020. In total, 13,991 nightingale song recordings were submitted to the project by anonymous (64%) and non-anonymous participants (36%). We achieved the goal of high community engagement, even though only a few non-anonymous citizen scientists participated in all three nightingale breeding seasons. Similar to other CS projects (e.g., Segal et al. 2015; Seymour and Haklay 2017), most of the non-anonymous participants took part in only a single nightingale breeding season. However, proportionately, these 1-year citizen scientists produced many and anonymous ones produced the majority of recordings. Anonymity may have been a success factor, without which two thirds of the data would not have been obtained. Bryant and Oliver (2009) demonstrated that dissemination activities do not seem to be a contributor to success for the duration or community engagement of participation in CS projects. Similarly, we assume that some citizen scientists liked joining our diverse dissemination activities but rarely generated recordings, while others recorded but did not attend. Even though press coverage and events did not seem to have an influence on the data scope (i.e., number of citizen scientists, duration and number of recordings), their reduction during the COVID-19 outbreak led to fewer participants and possibly shorter recording duration than in the previous years. These fewer citizen scientists in 2020 were individually more engaged and produced more recordings. The enhanced use as well as the improvement of the PRA may have led to an increase in the number and a decrease in the duration of recordings in the last year. Our findings are also consistent with other CS projects that reported higher individual engagement during the pandemic (e.g., Sánchez-Clavijo et al. 2021; Hochachka et al. 2021) based presumably on an increased desire to experience nature (Flaccus 2020) or outdoor activities (Venter et al. 2021). The geographical spread of our opportunistic data increased over the course of the project and was similar to the nightingale distribution in Germany (Gedeon et al. 2014). Moreover, our project was successful enough to expand the initial geographical focus from Berlin to a nationwide data coverage.

The low-threshold approach which engaged a wide audience of citizen scientists was also successful in this project. Contrary to Falk et al. (2019), participants as a mass may have improved, resulting in higher data accuracy than in other CS projects (e.g., Kosmala et al. 2016). In fact, the data quality of anonymous users was similar to those who participated for multiple years non-anonymously. This revealed that quality was not negatively affected neither by reduction of our dissemination activities (Bryant and Oliver, 2009) nor by anonymity of participants (Dickinson et al. 2010) but positively influenced by the project duration and the improved PRA.

### Lessons learned: suggestions for future CS projects on birdsong

As there is evidence in the literature that in many cases, data quality is linked to avoidable errors in the study design (e.g., Bowser et al. 2020), we suggest the following measures for future CS projects:

Most species misidentifications did not occur at night, as expected, but were affected by vocal similarities of other species. We should, for example, have provided more information through the use of the already integrated 'Species Portrait' and 'Trait Selection' features of the app, which could have reduced confusions with other species based on vocal similarities. Citizen scientists should have been supported with more specific instructions for the recordings, i.e., how (smartphone orientation), where (regions), and when (during the night). Such a task-lead approach facilitates data collection for citizen scientists (Moczek et al. 2021) and enables them to get a common understanding of data quality (Land-Zandstra et al. 2021). Positive feedback and confirmation of the number of successfully recorded nightingale songs likewise might have helped to increase quality (Peltola and Arpin 2018) and motivate citizen scientists to participate long-term (Pandya and Dibner 2018). It has been suggested that rewards (Reeves et al. 2017), greater involvement in scientific processes and valuing individual contributions (Dowthwaite and Sprinks 2019) could lead to data generated not by a few participants, (90-9-1 rule: Gasparini et al. 2020), but by many and further over the whole project duration. In future projects, the aspects that influence data quality could be validated and specified by additional demographic data (e.g., leisure activities; Lee and Scott 2004). Equally informative would be a self-assessment item or a scale of skills and knowledge to compare the recordings (type, number and duration).

Overall, the project was very successful and has positively evolved over the years. As individual engagement, data scope, spatial distribution, and data quality have largely increased over the nightingale breeding seasons, this strongly suggests that CS projects need to be implemented over the long term. It also became apparent that it was indeed a good idea to focus on a specific species like the nightingale, since species identification is a difficult task (Crall et al. 2011) and the nightingale proved to be a charismatic focal bird.

## Conclusion

The nightingale citizen science project has demonstrated that CS is a research approach that can contribute large datasets with data quality valid to science through increasing community development and engagement over time. For dialect research, many and diverse recordings from various locations are necessary, making our CS data highly valuable. The findings from our study may also offer great value for other CS projects to gain insights into best practices and to avoid systematic species misidentifications in the future, which can lead to biased ecological estimates (Dickinson et al. 2010). In sum, our study may inspire other existing and evolving CS projects in ornithology to adapt their study design with regard to their modes of community engagement and ways to ensure data quality.

## Supplementary Information

Below is the link to the electronic supplementary material.

Supplementary file1 (XLSX 10 KB)

Supplementary file2 (XLSX 10 KB)

## Data Availability

The datasets generated during and/or analysed during the current study are available from the corresponding author on reasonable request.
